# Optimization of Laser Cladding Process Parameters and Analysis of Organizational Properties of Mixer Liners

**DOI:** 10.3390/ma17215158

**Published:** 2024-10-23

**Authors:** Renwei Jiang, Chaosen Lin, Yuedan Li, Cuiyong Tang, Xueyong Chen

**Affiliations:** 1 College of Mechanical and Electrical Engineering, Fujian Agriculture and Forestry University, Fuzhou 350001, China; 1221213004@fafu.edu.cn (R.J.); dingdeng1224@163.com (C.L.); hnrtcy@163.com (C.T.); 2 School of Mechanical Engineering and Automation, Fuzhou University, Fuzhou 350001, China; 240210020@fzu.edu.cn

**Keywords:** laser cladding, surface strengthening, weighted and comprehensive evaluation, wear resistance

## Abstract

Aiming to address the wear and replacement inconvenience of concrete mixer liners, this study utilizes a laser cladding system to clad Fe60 alloy powder on the liner. It investigates the influence of different process parameters on the forming quality of the Fe60 alloy powder cladding layer. The optimal process parameters were obtained by weighted comprehensive evaluation, and single-layer multi-pass cladding experiments were carried out under the optimal process parameters to investigate the effects of a 30%, 40%, and 50% lap rate on the surface flatness and forming quality of the cladding layer. Using a metallographic microscope, a scanning electron microscope analysis of the macro morphology and microstructure of the cladding layer was conducted, a DPT-5 penetration flaw detector was used to observe the cracks on the surface of the multi-channel cladding, a microhardness tester and friction and wear experimental machine were used for the hardness of the cladding layer, and an abrasive wear resistance test was conducted. The results show that under the process parameters of a laser power of 900 W, powder feeding speed of 7 g/min, scanning speed of 600 mm/min, and 50% lap rate, the average microhardness of the fused cladding layer reaches 742 HV, which is 1.8 times higher than that of the liner plate, and the coefficient of friction is 0.57, which improves the liner plate’s wear resistance performance and service life.

## 1. Introduction

The mixer liner is a key component of the concrete mixer, and its main role is to protect the mixer cylinder from the direct impact and abrasion of the abrasive body and materials, thereby prolonging the service life of the cylinder and improving mixing efficiency and output. However, the mixer liner faces a harsh working environment, with not only having to withstand abrasive wear but also needing to resist the corrosive wear of slurry, with abrasive wear being the main cause of failure [1]. The frequent replacement of liners not only increases maintenance and utilization costs but reduces production efficiency, leading to serious economic losses. Therefore, enhancing the wear resistance of mixer liners significantly extends service life, improves production efficiency, reduces maintenance burdens, and saves resources. This comprehensive economic benefit makes research on liner wear resistance particularly urgent and important.

As an advanced surface treatment technology, laser cladding technology has the advantages of a high energy density, controllable dilution rate, fast processing speed, and small heat-affected zone [2]. Laser cladding technology can form a good metallurgical bond between the coating and the substrate to ensure the bond strength between the coating and the substrate [3]. The resulting cladding layer protects the substrate components from external wear and corrosion and improves other surface properties of the substrate [4]. Shuai Zhang et al. [5] used the laser cladding of an iron-based wear-resistant alloy to strengthen a seamless straightness gauge roller’s surface wear resistance. The results show that the laser cladding-reinforced layer forms a solid metallurgical bond with the roller substrate. The average width of the abrasion mark in the 30 min sliding friction wear test was only 1.43 mm, and the abrasion resistance of the cladding reinforced layer was about 24% higher than that of the quenched reinforced layer. Wang [6] et al. prepared Ni60 + 30%WC composite coatings on the surface of Q235 steel by varying the parameters of the different laser cladding processes, and the study showed that the scanning the scanning speed had a greater influence on the adjustment of the aspect ratio and dilution rate of the cladding layer, especially in affecting the forming quality more significantly than the effect of laser power. Under appropriate process parameters, the microstructure of the coating is denser and more uniform, and the hardness and wear resistance are significantly better than that of the substrate. Liu Zhiling [7] et al. used laser cladding to prepare Ni60 + TiC gradient layer structure coatings on the surface of 45 steel and investigated the effect of process parameters on the organization and properties of the coatings. It was found that the microhardness of the coatings was a gradient from 1968 HV0.2 to 240 HV0.2 by the table under the optimum process parameters, and the wear rate of the coatings was 1.757 × 10–15 m^3^/(N·m).

It has been found that the laser cladding process parameters greatly influence the quality of the cladding layer, among which the process parameters with significant influence are laser power, scanning speed, powder feeding speed, etc. [8]. At present, most of the optimal process parameters for laser melting are selected through the analysis of extreme deviation and the analysis of variance through orthogonal tests [9,10,11], and the optimal process parameters are less frequently obtained using the weighted comprehensive evaluation in the current study. Therefore, in this study, the optimal process parameters for laser cladding were selected using a weighted comprehensive evaluation, and high hardness wear-resistant iron-based coatings were prepared on the surface of a mixer liner using laser cladding technology, the organizational distribution law of the cladding layer was analyzed through numerical simulation, and the interrelationships between the microstructure and mechanical properties of the coatings were investigated in depth. The aim was to improve the wear resistance performance of the mixer liner and extend its service life to provide a solid theoretical basis.

## 2. Test Materials and Methods

### 2.1. Test Materials

In this study, a grade JS500 concrete mixer liner (Zhuhai Scoma Machinery Equipment Co., Zhuhai, China) was used as the object of study. Its main chemical composition is as shown in Table 1.

**Figure 1:**
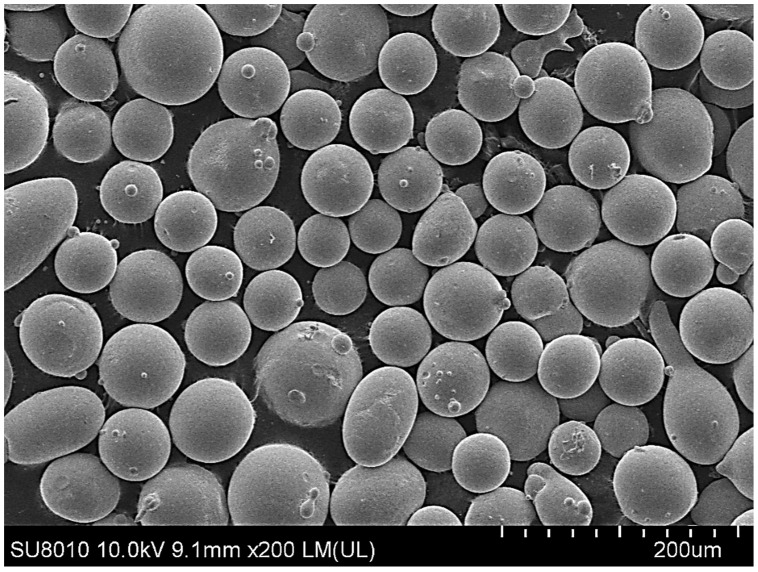
Microstructure of Fe60 alloy powder.

For Fe60 alloy powder, based on its material properties, its composition and coefficient of thermal expansion are similar to those of the mixer liner to avoid excessive residual stress in the fusion cladding layer, reduce cracks, and produce good metallurgical bonding. At the same time, Fe60 alloy powder is a self-soluble alloy powder, so that it maintains good wetting with the substrate [12]. In addition, Fe60 alloy powder also has high wear resistance, corrosion resistance, and a low price, which is suitable for strengthening the liner, such as in occasions with a high area and high powder demand. This study used the Fe60 alloy powder from Qinghe County Casting Gold Welding Materials Co., Ltd. (Xingtai City, China). Its particle size is 45–106 μm, as shown in Figure 1, for the Fe60 alloy powder microscopic morphology, and its chemical composition is shown in Table 2.

### 2.2. Test Methods

Before experimenting, the substrate needs to be pre-treated by grinding the surface of the substrate with an abrasive wheel and then using 600 # sandpaper to remove the oxide layer on the surface. Ultrasonic cleaning with anhydrous ethanol dissolves and removes stubborn impurities such as oil, rust, etc. to ensure that the cleanliness of the substrate meets the experimental requirements. Finally, the substrate is dried and stored in a DZF-6090 vacuum drying oven. At the beginning of the experiment, it can be taken out and used directly.

This study utilized the laser cladding equipment produced by Dazu Laser Science and Technology Industry Group to prepare laser cladding coating on the surface of a concrete mixer liner, and the system mainly consists of an HL-WM-4000 laser cladding machine, cooling system, powder feeder, voltage stabilizer, pure argon protective gas, and computer control system. The laser wavelength used in the experiment was 1080 nm, and the main parameters of the laser cladding equipment are shown in Table 3.

The middle area of the laser fusion coating was selected, and the specimen was cut along the direction perpendicular to the coating using a wire cutter. The cut section was polished step by step using 160 #, 320 #, 600 #, 1200 #, 1500 #, and 2000 # sandpaper, and finally polished with an abrasive paste with a 2000-mesh grain size on a grinding and polishing machine. The polished specimens were corroded using aqua regia, followed by cleaning the residual corrosion solution and drying the specimens. A preliminary observation of the microstructure of the specimens was carried out using a DM2700M metallurgical microscope. The microstructure of the coating was observed using a high-precision scanning electron microscope model SU8010 produced by Hitachi (Chiyoda City, Japan).

This test selects the DPT-5 penetration flaw detector as a tool; the flaw detector includes three parts: a cleaning agent, a penetrant, and a developer. The operation steps are as follows: first of all, use the cleaning agent to clean the specimen, and then, in a dry ventilated place, make the surface completely dry to ensure that the test results are not affected by moisture or other impurities; then, spray penetrant on the surface of the coating and let it stand for 15 min in order to form a stable wet layer; then, use a cleaning agent to remove excess penetrant on the surface of the coating so as not to interfere with the subsequent observation; then, maintain a certain spacing for uniform spraying; then, evenly spray the developer on the surface of the coating at a certain spacing and leave it for about 5 min to make it react with the defects inside the coating and show them; and finally, carefully observe the surface of the specimen to see whether cracks appear.

Use an HMV-10S type Vickers microhardness tester to measure the microhardness on the cross-section of the fusion coating, under the parameters of a load of 1000 g and loading time of 10 s to measure the microhardness of the coating, using a CCD (Charge-Coupled Device) to collect the specimen on the diamond indentation produced by the diamond over the area to determine the degree of microhardness, the direction of hardness measurements along the fusion cladding layer on top of the heat-affected zone, and the substrate in the order of the sequential measurements; the distance between measurement points was 0.15 mm. 

Reciprocating friction experiments were carried out using the RTEC-MFT5000 multifunctional friction and wear tester to obtain the coefficient of friction of the specimen and the parameters of the abrasion marks by setting up specific parameters to make the coating and the friction sub-materials move relative to each other and thus to generate the opposing abrasion. In the experiment, the selected material for the counter-abrasion was an Al_2_O_3_ ceramic ball, a loading load of 10 N was applied to the coating surface to run a 5 mm abrasion track, the frequency was set to 6 HZ, and the loading was continued for 40 min in order to observe and record the abrasion process. Immediately after completing the test, ultrasonic cleaning with anhydrous ethanol was carried out for 15 min to ensure that residues were adequately removed. Subsequently, the specimens were dried in a desiccator until completely dry. Then, the mass loss of the samples was accurately measured using a precision electronic scale. The above operation was repeated for three friction experiments to take the average value, which was used as a reference for subsequent analyses.

### 2.3. Finite Element Modeling

Laser cladding is a fast and complex transient process, and the following reasonable assumptions need to be made in order to facilitate the simulation of the laser cladding process and to improve the computational efficiency:

(1) The powder coating and the base material are uniformly continuous and isotropic;
(2) Thermal energy loss due to material vaporization is neglected during laser cladding;
(3) Thermo-physical parameters of materials such as density, specific heat capacity, thermal conductivity, etc., are only temperature-dependent, while other material parameters are constants;
(4) The impact of protective gas and metal powder on the coating morphology is neglected during the cladding process;
(5) The fluid in the melt pool is Newtonian incompressible and laminar;
(6) Physical phenomena such as scattering and the refraction of lasers are neglected.

**Figure 2:**
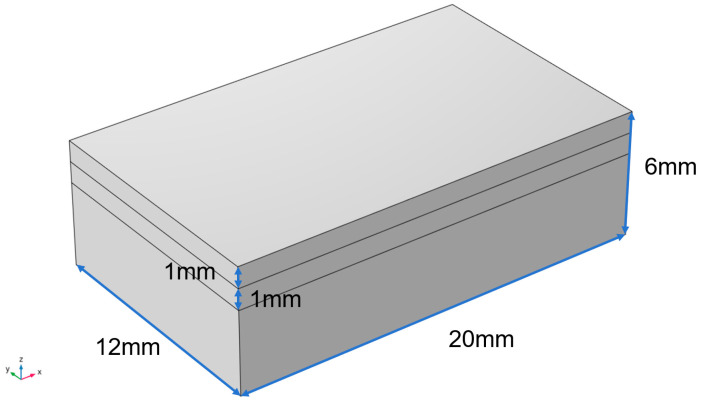
Geometric model.

In this study, the built-in geometry module of COMSOL Multiphysics was used to establish a finite element geometric model for laser cladding with an overall size of the substrate of 20 mm by 12 mm by 6 mm, as shown in Figure 2.

**Figure 3:**
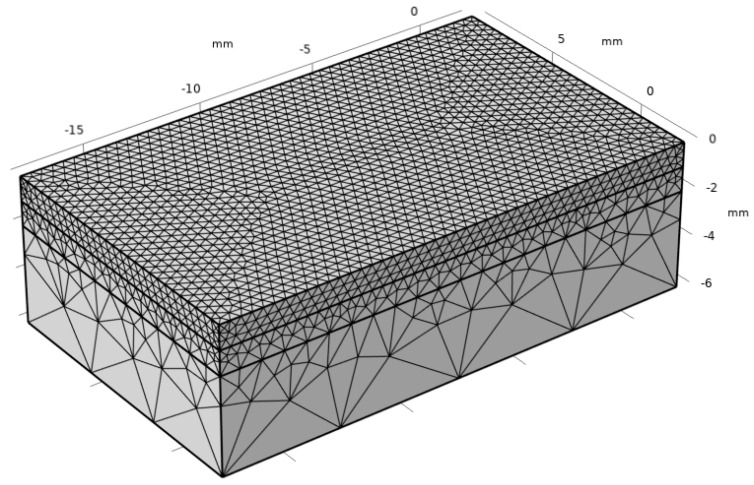
Mesh division of the laser cladding.

The geometric model is divided into three parts: the upper layer is the cladding region; in order to improve the calculation accuracy, this part of the mesh is fine. The lower layer is far from the cladding region; in order to improve the calculation efficiency, this part of the mesh is extremely coarse. The middle mesh adopts the normal mesh, which is used to make the mesh smooth transition, as shown in Figure 3. The specific grid size parameters are shown in Table 4.

The temperature field modeling of the laser cladding process incorporates three basic modes of heat transfer: heat conduction, heat convection, and heat radiation [13,14].

(1) Heat conduction

In all fields, the heat transfer equation is

(1) $$\rho c_{p}\left(\frac{\partial T}{\partial t}+u\nabla T\right)=\nabla (\lambda \nabla T)$$

which is solved in terms of density ρ, specific heat Cp, time t, and thermal conductivity λ.

(2) Heat convection

The heat convection equation between the boundary of the model and the surroundings is

(2) $$Q_{\mathrm{convection}}=h_{c}\left(T-T_{0}\right)$$

where the initial temperature is $T_{0}$, and $h_{c}$ is the convective heat transfer coefficient.

(3) Heat radiation

The heat radiation equation between the model boundary and the surroundings is

(3) $$Q_{\mathrm{diffuse}}=\sigma \varepsilon \left(T^{4}-T_{0}^{4}\right)$$

where σ is the Stephen–Boltzmann constant, and ε is the surface heat dissipation rate.

The Gaussian heat source model [15] was used for the laser heat source model in this study with the following expression:

(4) $$Q_{\mathrm{laser}}=\frac{2\alpha (1-\beta )P}{\pi {r_{0}}^{2}}\exp\left\{-\frac{2({\left(x-v\cdot t\right)}^{2}+y^{2})}{{r_{0}}^{2}}\right\}$$

where the laser absorption rate is α; the laser masking rate is β; P is the laser power; $r_{0}$ is the spot radius; and v is the scanning speed.

In order to better simulate the macroscopic morphology of the fusion cladding layer, the Moving Mesh in the COMSOL Multiphysics (6.0) software was used to achieve the stacking and molding of the melted powder on the surface of the substrate, and the moving speed equation of the dynamic mesh at the fusion cladding interface [16] was set as follows:

(5) $$V_{z}=\frac{\varphi \tau }{3\pi r_{m}^{2}}\exp\left\{-\frac{2({\left(x-v\cdot t\right)}^{2}+y^{2}}{r_{m}^{2}}\right\}$$

where the powder feed speed is φ, τ is the powder feed speed coefficient measured based on a large number of previous laboratory studies, and $r_{m}$ is the radius of the powder flow.

The thermo-physical parameters of the mixer lining plates and Fe60 alloy powders, as well as the values of the detailed parameters used in the calculations, are shown in Table 5, Table 6 and Table 7.

### 2.4. Relevant Definitions

#### 2.4.1. Weighted Comprehensive Evaluation

The weighted comprehensive evaluation method provides a more comprehensive and objective result by combining information from multiple indicators. This method sets different weighting coefficients based on the relative importance of each indicator in the overall evaluation. Specifically, the more important the indicator is in the overall evaluation, the higher its weighting factor, and conversely, the lower the weighting factor. The steps of the method include assigning weighting coefficients to the indicators according to their hierarchical importance, then scoring the indicators of the object to be evaluated, and finally combining the weighting coefficients with the scores of the indicators to arrive at an objective and comprehensive evaluation result.

#### 2.4.2. Dilution Rate

**Figure 4:**
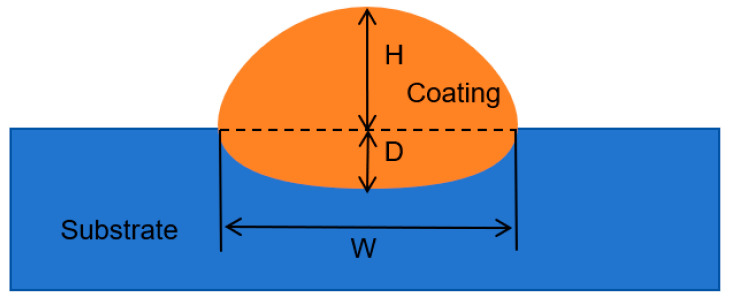
Dilution rate calculation schematic.

The dilution rate refers to the laser cladding process after melting the base material and mixing the cladding material, resulting in changes in the composition of the cladding alloy, which is one of the important indicators for evaluating the quality of the cladding layer. A high dilution rate tends to significantly reduce the performance of the cladding layer, making it more prone to cracking and deformation; on the contrary, if the dilution rate is too low, it will lead to a weak bonding strength between the substrate and the cladding layer, which will make the cladding layer fall off. In this study, by measuring the melting height *H*, the melting depth of the value *D*, according to the calculation of Formula (6) [17], can be obtained dilution rate, as shown in Figure 4, where *H* is the height of the fused cladding layer, *D* is the depth of melting of the substrate material, and *W* is the width of the fused cladding layer.

(6) $$\eta =\frac{D}{H+D}\times 100\%$$

#### 2.4.3. Overlap Rate

**Figure 5:**
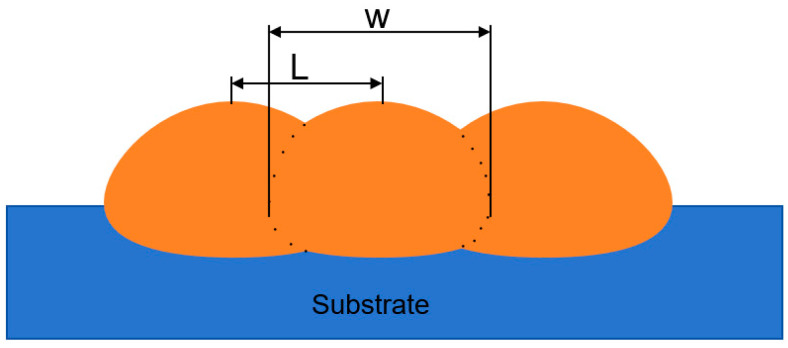
Schematic of multi-pass overlap rate cross-section of cladding coating.

The overlap rate refers to the overlapping area between two adjacent single passes of laser coating in the process of laser melting, which is the key parameter for controlling the multi-pass lap laser melting process, as shown in Figure 5, for the cross-sectional morphology of the multi-pass lap rate of the melting coating.

The lap ratio φ is the ratio between the offset L and the melt width W [18], as shown in Equation (7):

(7) $$\varphi =\frac{L}{W}$$

## 3. Optimization of Laser Cladding Process Parameters and Analysis of Their Organization and Properties

### 3.1. Optimization of Laser Cladding Process Parameters

#### 3.1.1. Orthogonal Experimental Design

The quality of single-pass laser fusion coating is an important indicator of single- or even multi-layer fusion cladding. In this study, a three-factor, three-level L_9_(3^4^) orthogonal test was used to select the values of the levels of each factor, as shown in Table 8, and the design of the orthogonal test table is shown in Table 9.

#### 3.1.2. Weighted Comprehensive Evaluation and Evaluation Criteria

Hardness is regarded as an important indicator of a material’s wear resistance, and it has been found that hardness is usually positively correlated with wear resistance [19]. The dilution rate affects the composition, organization, and bonding strength between the coating and the substrate, with too low a dilution rate resulting in a weak bond between the coating and the substrate, and too high a dilution rate affecting the coating performance [20]. Under the precondition of ensuring the metallurgical bonding between the coating and the substrate, the appropriate dilution rate is conducive to stabilizing the composition and organization of the designed coating. Surface quality affects the precision of the assembly quality and is also an important indicator. According to the actual application in the mixer liner, the expectations for the fusion coating on top of the mixer liner are in the following order: high hardness > dilution rate > coating surface quality. According to each evaluation index’s different degrees of importance, the three evaluation indexes are first given the weight coefficient Q and then evaluated and scored N. The comprehensive score T is calculated, and the best process parameters are finally selected. The formula for calculating the comprehensive score is $T=\sum Q_{i}\times N_{i}$, where i = 1, 2, 3.

In this multi-objective optimization, the weights are taken as follows: hardness weight is taken as 5, dilution rate weight is taken as 3, and the surface quality of the cladding layer is taken as 2. The scoring standard of microhardness is shown in Table 10, the scoring standard of surface quality of the cladding layer is shown in Table 11, and the scoring standard of the dilution rate is shown in Table 12, which is scored according to the indexes of superiority and inferiority, and the range of scoring is from 1 to 10.

#### 3.1.3. Optimization Results

Table 13 shows the macroscopic and cross-sectional microscopic morphology of nine groups of single-pass fused cladding layers obtained from orthogonal tests. The macroscopic morphology of all fused cladding layers was 12 mm in length, and the magnification of the microscopic cross-section morphology was the same.

The study results found that the laser energy was mainly concentrated on the fused cladding powder for the specimens with lower laser power. When the powder starts to melt, the remaining laser energy is not enough to bring the substrate to the melting state, which makes the dilution rate of the fused cladding layer very low, and this melting process results in low bonding strength between the substrate and the fused cladding layer, leading to a decrease in the overall performance of the fused cladding layer, as shown in specimens 1, 2, and 3.

The main reason for the shallow molten pool in specimen 5 is the increase in the powder feeding rate and the scanning speed, which leads to an increase in the amount of powder sprayed on the surface of the substrate per unit of time, resulting in a higher height of the cladding layer than in other specimens, but the laser energy that reaches the surface of the substrate is extremely limited and therefore does not lead to the formation of a sufficiently large molten pool in the substrate material. When the amount of powder delivered is too large or the scanning speed is low, it leads to a large wetting angle in laser cladding. When the wetting angle is large, defects such as unfused and inclusions are likely to occur in multi-layer, multi-pass cladding [21].

The coating of specimens 4 and 7 did not show pores, cracks, or the surface residue phenomenon, but due to the scanning speed being small and the powder feeding rate being large, there was more substrate surface coating powder, resulting in a large wetting angle, which is not conducive to the later multi-pass laser coating. The cross-section topography quality of specimens 6, 8, and 9 was good, with no cracks or large wetting angle phenomenon.

In this study, the indentation method was used to measure the microhardness of the fused cladding cross-section, and the polished and corroded fused cladding specimens were tested point-by-point along the top of the fused cladding layer in the substrate direction. The calculated values of the specimen dilution rate and microhardness are shown in Table 14.

Table 15 shows the results of the weighted comprehensive analysis. It can be seen from the table that the No. 6 specimen had the highest comprehensive score, indicating that the process parameters can well meet the expectations of a high microhardness of the coating, moderate dilution rate, and good surface quality of the cladding layer. It can be seen that the best single-pass laser cladding process parameters are a laser power of 900 W, powder feeding speed of 7 g/min, and scanning speed of 600 mm/min.

### 3.2. Selection of Lap Ratio for Single-Pass Fused Cladding Layer

Due to the limited width of single-pass laser cladding, it is difficult to meet the actual needs of the industry at present. Therefore, it is necessary to realize the reinforcement of the mixer liner surface in a large area by means of multi-pass laser cladding.

**Figure 6:**
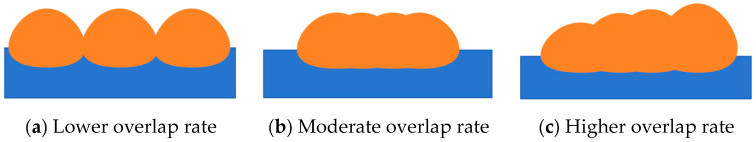
Cross-section morphologies of cladding layers at different overlap rates.

The lap rate of multi-pass cladding has an important influence on the surface flatness of laser cladding coatings and the preparation of high-quality coatings [22]. When the overlap rate is too low, obvious grooves will appear between neighboring coatings, resulting in an uneven surface and increasing the subsequent processing cost; when the overlap rate is too large, the coatings will be highly overlapped, and the height of one side of the cladding is higher than the other, resulting in thickness variation and material waste, and may cause deformation of the substrate due to the increase in energy absorption. Figure 6 shows the cross-sectional morphology of the fusion-coated layer under different lap rates. Therefore, selecting an appropriate lap rate is critical to ensure the best balance between coating quality and forming effect.

Based on this group’s previous research, three coatings with different lap rates were laser-melted onto the surface of the mixer liner. In order to obtain accurate comparative data, three sets of laser cladding tests with different lap rates were carried out under the same optimal process parameters and scanning trajectories. The specific process parameters of the cladding layers with different lap rates are shown in Table 16.

**Figure 7:**
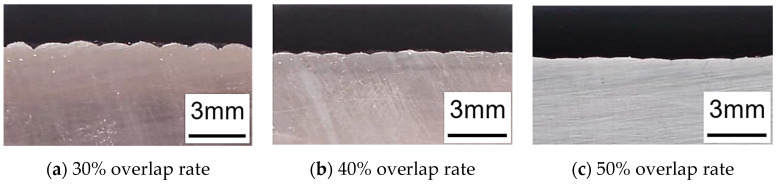
Cross-section morphologies of cladding layers at different bonding rates.

Figure 7 shows the cross-sectional morphology of the fusion-coated layer under different lap rates. As can be seen from the figure, when the lap rate is 30%, the surface lap area of the Fe60 alloy coating is very obviously concave, and the surface is not flat. When the lap rate is 40%, the surface lap area of the Fe60 alloy coating is still concave, but the concave area has been alleviated to some extent. When the lap rate is 50%, the surface lap area of the Fe60 alloy coating becomes flat, and the lap area becomes smooth and uniform, with good overall morphology.

**Figure 8:**
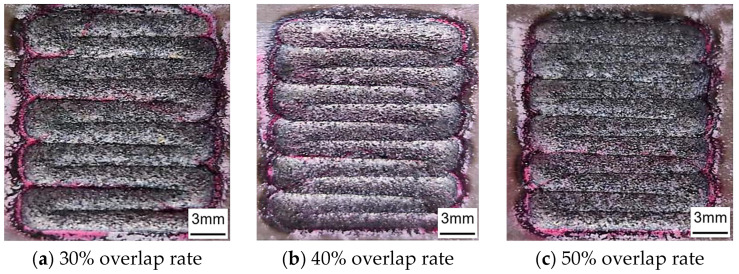
Crack detection of cladding coatings with different overlap rates.

According to scholars’ research, the multi-pass laser cladding lap region is prone to residual stresses, which can easily lead to cracks in the coating [23]. Whether or not cracks occur in the lap rate region is one of the important criteria for determining the quality of laser cladding coatings. Therefore, in this study, surface coloring flaw detectors were used to detect cracks in laser cladding coatings. As shown in Figure 8, no cracks were observed in the three groups of fused coatings with different lap rates, proving that the fused Fe60 alloy coating on the surface of the mixer liner has good formability.

According to the above analysis, from the careful consideration of the flatness of th Fe60 alloy coating and the overall morphology of the coating, when a 50% lap rate is selected for the preparation of a laser cladding coating on the surface of the mixer liner, it both has better flatness and does not produce cracks, so a 50% lap rate is the optimal choice.

### 3.3. Analysis of the Organization and Properties of Single-Pass Fusion Cladding Layer

#### 3.3.1. Organizational Analysis of the Cladding Layer

**Figure 9:**
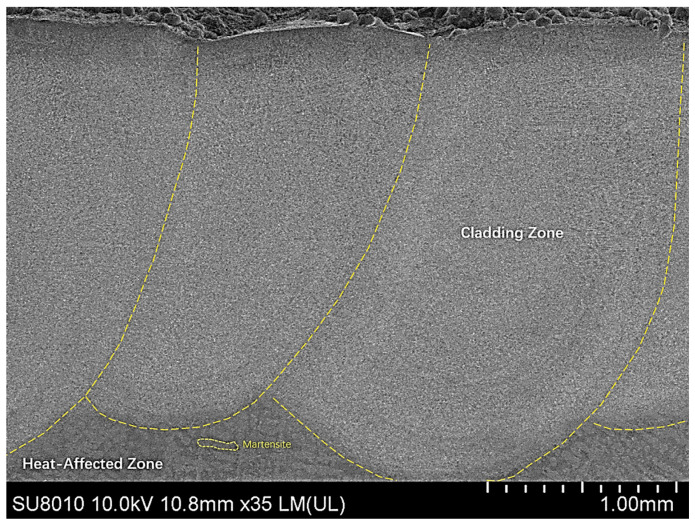
Macroscopic morphology of Fe60 alloy powder multi-pass fusion coating with 50% overlap rate.

Figure 9 shows the macroscopic morphology of an Fe60 alloy powder multi-laser coating with a 50% overlap rate on the concrete mixer liner surface. As can be seen from the figure, the multi-pass laser cladding coating has good bonding in the overlap area without cracks, residues, or other defects, and the overlap area is smooth and uniform. The bond between the coating and the substrate formed a certain thickness and dense planar crystals, which is an important basis for the formation of a solid metallurgical bond between the substrate and the cladding coating [24]. In the heat-affected zone near the bottom of the coating, many needle-like and flaky martensitic structures are visible. This phenomenon is due to the action of the laser beam, which causes the substrate material close to the coating to rapidly heat up to the austenitizing temperature. After the laser beam is removed, the temperature in this region decreases rapidly, which triggers a partial austenite to martensite transformation.

**Figure 10:**
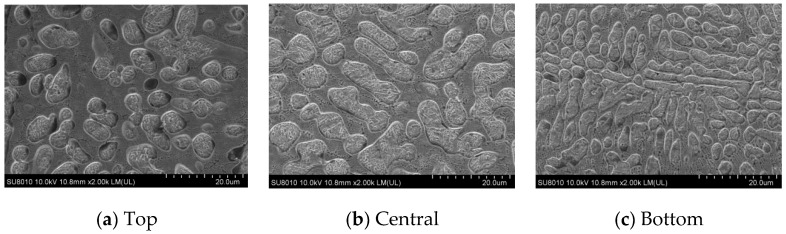
Microstructure and morphology of laser cladding coatings under the optimal process.

Figure 10 shows the organization of the laser cladding coatings under the optimum process. The microstructure of the coatings shows coarse cytosolic/columnar crystals, disordered and dense dendritic crystals, and equiaxed crystals from the bottom to the top, respectively.

**Figure 11:**
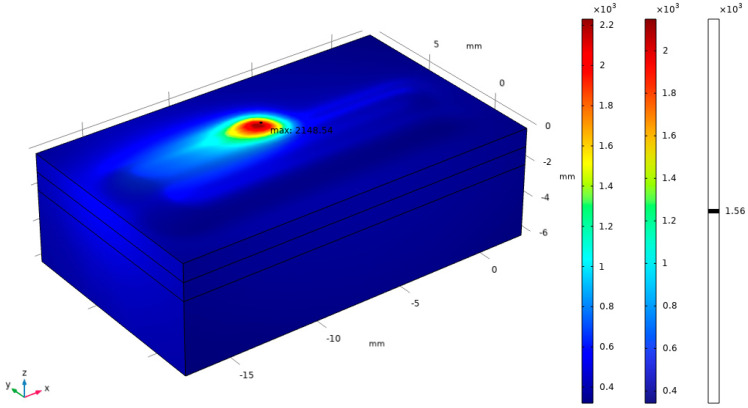
Cloud diagram of temperature distribution of single-layer multi-pass laser cladding.

Based on rapid solidification theory, the tissue morphology formed during laser cladding is mainly determined by the temperature gradient (G) and solidification rate (R) of the liquid phase along the solid–liquid interface [25]. The solidified tissue growth morphology in the melt pool is mainly controlled by the shape control factor: the ratio between G and R (G/R). In order to better study the effects of the temperature gradient and solidification rate on the tissue shaping of the molten cladding layer, as well as to provide a theoretical basis for the tissue distribution of the molten cladding layer, the present study utilized the COMSOL Multiphysics software to carry out a numerical simulation of the temperature field for the single-layer multi-channel cladding test. In order to reduce the amount of modeling calculations, a four-pass cladding simulation was carried out. Figure 11 shows a cloud diagram of the temperature distribution under the Gaussian heat source model, and the highest temperature of the melt pool is about 2148 K.

**Figure 12:**
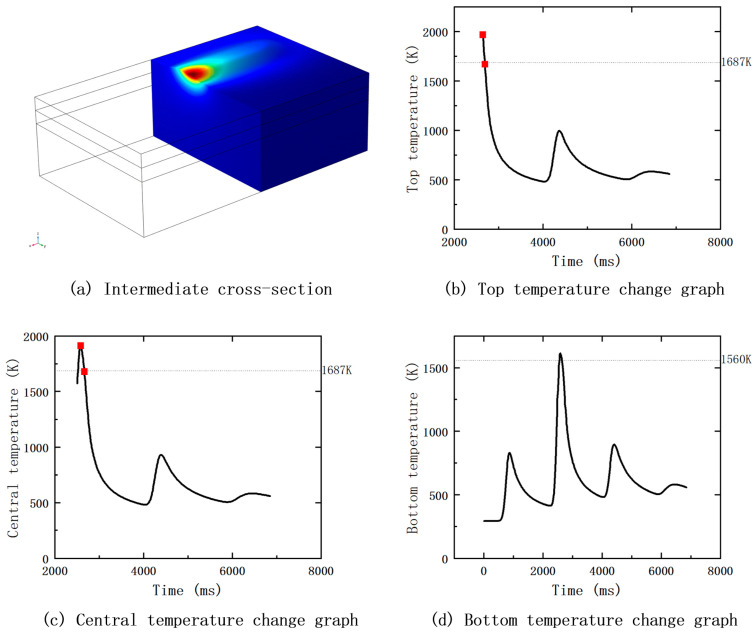
Temperature profiles of the top, middle, and bottom of the middle section of the fused cladding layer.

In this study, the top, middle, and bottom at the middle section of the second fusion cladding layer were analyzed, and the laser arrives at this position at 2550 ms, as shown in Figure 12a; the simulation results of the temperature field can be used to obtain the temperature versus time graphs at the three positions, and Figure 12b–d show the temperature curves at the top, middle, and bottom, respectively. In Figure 12b,c, the horizontal black dotted line represents the melting point of Fe60 powder: 1687 K. When the temperature drops from the highest point to the melting point, the temperature profile can be regarded as a straight line, i.e. the line segment between the two red squares in the figure. When calculating the slope of the line between the two red squares, this slope can be used as the average cooling rate at that location. The same method can be used for the bottom of the cooling rate, but at this time, the melting point for the melting point of the matrix is 1560 K.

The temperature gradient *G* can be obtained directly from the simulation results; for the solidification rate *R*, it needs to be obtained indirectly using Equation (8) [26], where is the cooling rate.

(8) ε=G×R

**Figure 13:**
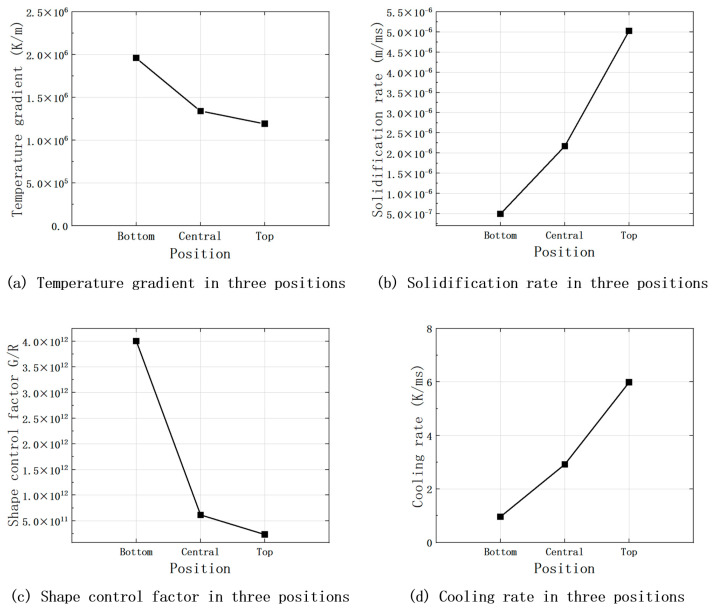
Solidification data related to the top, middle, and bottom of the fused cladding at the middle cross-section.

The temperature gradient *G*, solidification rate *R*, shape control factor *G*/*R*, and cooling rate of the three parts of the cladding can be obtained. As shown in Figure 13, the results show that from the bottom to the top of the cladding layer, the temperature gradient and shape control factor show a decreasing trend, while the solidification rate and cooling rate show an increasing trend.

**Figure 14:**
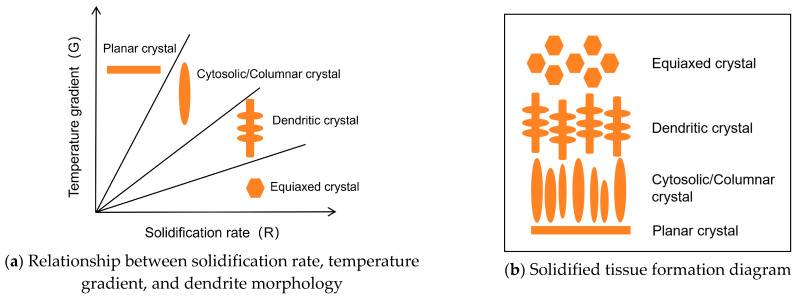
Relation and principle of dendrite morphology.

Figure 14a shows the relationship between G and R and the solidified tissue morphology with a schematic of the solidified tissue formation. In analyzing the results of Figure 13, at the bottom of the molten pool, where the cladding layer is in direct contact with the substrate and dissipates heat through the substrate, the G of the liquid phase at the solid–liquid interface is larger, while the R is smaller, resulting in the maximum value of G/R. In this case, there is no significant solidified tissue formation in the liquid phase at the solid–liquid interface. In this case, there is no significant “compositional subcooling” in the liquid phase at the solid–liquid interface front, so the solidified interfacial organization forms thin planar crystals. As the solid–liquid interface moves forward, G gradually decreases, R increases, and the value of G/R decreases accordingly, which increases the “compositional subcooling” and leads to the instability of the solidified interface, and the tissue growth morphology changes from planar crystals to coarse cytosolic/columnar crystals. The solid–liquid interface continues to move forward, G continues to become smaller, R continues to increase, the value of G/R continues to become smaller, and the tissue growth morphology changes from coarse cytosolic/columnar crystals to dendritic crystals. When the solid–liquid interface pushes to the top of the molten cladding, the melt pool transfers heat through radiation and convection heat dissipation through the air medium and thermal conductivity of the matrix, and G drops to its lowest point, while R peaks, resulting in the value of G/R reaching a minimum. At this time, the solid–liquid interface at the front of the liquid phase in the “component supercooling” phenomenon is the most significant, and the grains are free to grow, showing anisotropic equiaxed crystals morphology. The final result is the distribution of the organization of the cladding layer as shown in Figure 14b. By comparison, this is consistent with the distribution presented in Figure 9 and Figure 10, proving the model’s accuracy.

#### 3.3.2. Analysis of the Properties of the Cladding Layer

**Figure 15:**
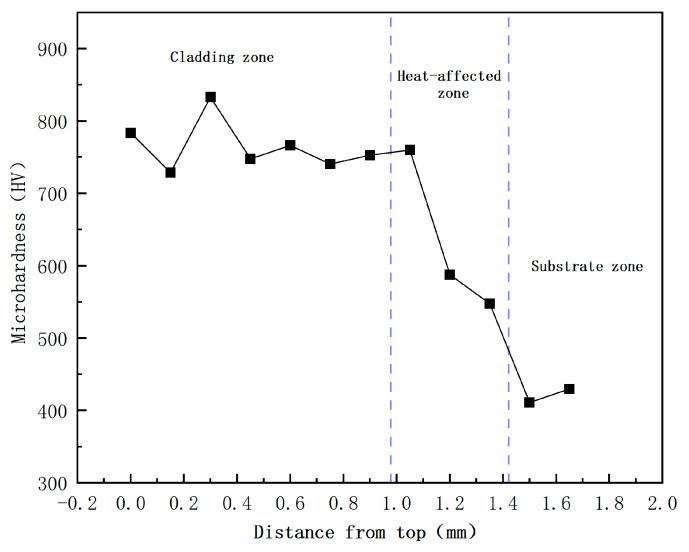
Microhardness distribution diagram of single-layer Fe60 powder cladding layer.

Figure 15 shows the point–line diagram of the microhardness distribution of the single-layer Fe60 alloy powder fusion cladding. The microhardness of the test cladding layer is divided into three regions: the cladding zone, the heat-affected zone, and the substrate zone. As can be seen from the figure, there is no sudden change in the microhardness of the three regions of the cladding layer. The average microhardness of the Fe60 alloy powder cladding layer is 742 HV, which is 1.8 times that of the substrate, which is mainly attributed to a few factors: Firstly, in the process of laser cladding, the alloying elements such as C, Cr, and other alloying elements polarized to form a high hardness and strength of the eutectic product [27]; secondly, the Cr element in the cladding layer can play the effect of solid solution strengthening, so that the microhardness of the Fe-based cladding layer has been improved; and finally, due to the characteristics of the laser cladding of fast heat and cold and the formation of dense and fine dendritic crystals and equiaxed crystals in the middle and the top of the cladding layer, as shown in Figure 10, the grains have been refined to induce the effect of fine crystalline reinforcement to a certain extent to improve the surface hardness.

**Figure 16:**
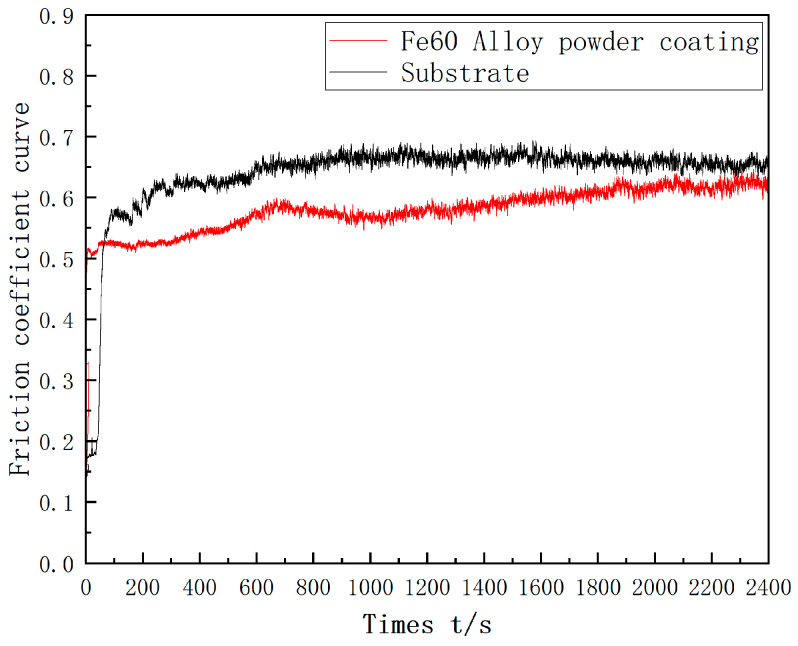
Friction coefficient curve of Fe-based cladding layer and matrix.

Figure 16 shows the friction coefficient curve of the single-layer Fe60 alloy powder cladding layer and the substrate. From the figure, it can be seen that the friction coefficient curve can be divided into a running and stabilizing stage, where the average friction coefficient of the molten cladding layer is 0.57, and the average friction coefficient of the substrate is 0.64. In the early running and stage, due to the surface of the molten cladding layer, there will be a small portion of the raised area, in the friction vice contact with the raised coatings, which makes the contact area of the friction vice increase, and the friction coefficient curve fluctuate, and after 4 min, it enters the stabilization stage. In general, the higher the microhardness of the material, the lower the coefficient of friction, which is consistent with the pattern exhibited by single-layer Fe60 alloy powder cladding. The coefficient of friction of the cladding layer is lower than that of the substrate due to the microhardness.

**Figure 17:**
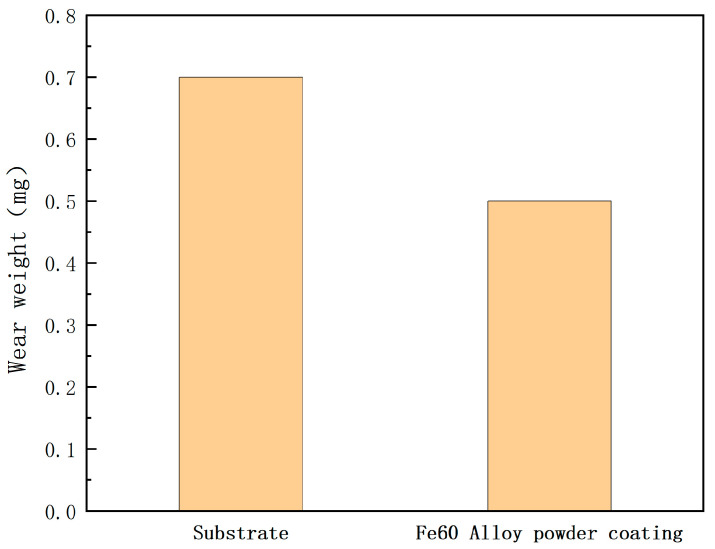
Wear amount of Fe60 powder cladding layer and matrix.

Figure 17 shows the wear mass of the single-layer Fe60 alloy powder fusion cladding layer and the substrate. As can be seen from the figure, the wear mass of the substrate and the single-layer Fe60 alloy powder cladding is 0.7 mg and 0.5 mg. After the surface reinforcement of the mixer liner, the wear mass increases to 1.4 times that of the substrate. Under the same friction conditions, the wear resistance of a material is inversely proportional to its wear mass; therefore, the laser-melted Fe60 alloy powder cladding on the surface of the mixer liner shows better wear resistance. The reason for the higher wear resistance of the Fe60 powder cladding layer compared with the substrate is that, on the one hand, its coefficient of friction is lower, which reduces the wear in the friction process; on the other hand, the cladding layer contains hard phases, forming a “soft and hard” structure, which protects the substrate in the friction and wear experiments and improves the resistance to cutting and deformation.

**Figure 18:**
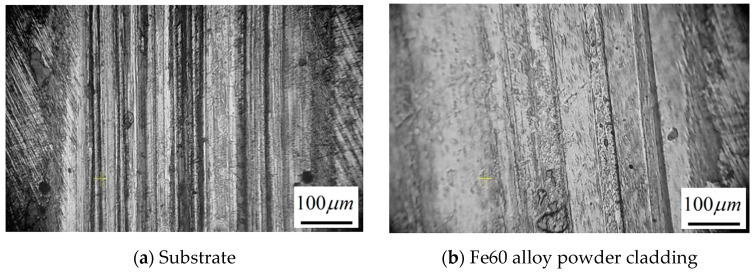
Surface wear morphology of the matrix and Fe60 alloy powder cladding layer.

The wear morphology of the single-layer Fe60 alloy powder fusion cladding layer and the substrate is shown in Figure 18. As can be seen from the figure, under the same dry friction conditions, the abrasion marks on the surface of the substrate appear as deeper and wider furrows, and the surface has pits and abrasive debris flaked off in the friction process. The main reason for this situation is that the microhardness of the substrate is smaller than the hardness of the friction sub-material, and the temperature generated in the friction and wear process will gradually increase, resulting in the softening of the surface material, and the metal in contact between the substrate and the friction sub-material will be adhered to in this situation. And in the friction process under the action of the alternating contact stress, the resulting abrasive debris will lead to fatigue cracks on the surface of the substrate, which will then make the abrasive debris show flaking; the main type of wear of the substrate is adhesive wear. The single-layer Fe60 alloy powder fusion cladding surface also appears in localized coating spalling, but the depth and size are smaller than those of the substrate, and the overall wear situation will be slighter. Because the microhardness of the Fe60 alloy powder fusion cladding layer is relatively high, in the friction wear process, the shedding of the hard phase can play a lubricating effect to a certain extent, and the hard phase in the fusion cladding layer also plays a role in the protection of the coating and the role of load bearing. The main type of wear of the single-layer Fe60 alloy powder cladding is adhesive wear.

## 4. Conclusions

In this study, Fe60 alloy powder was selected as the cladding material to strengthen the wear resistance of mixer liners. The weighted comprehensive evaluation was used to explore the optimal process of single-pass laser cladding on the surface of the mixer liner and to study the effect of lap rate on the quality of the cladding layer under the optimal single-pass cladding process parameters. The microstructure and mechanical properties of the single-layer cladding were also analyzed under the optimum lap rate. The following conclusions were obtained from this study:

(1) For the single-pass laser cladding of mixer liner surfaces, a laser power of 900 W, powder feed rate of 7 g/min, and scanning speed of 600 mm/min are the optimum process parameters. In this case, the single-pass laser cladding coating has a dense organization, producing a strong metallurgical bond with the substrate.

(2) A certain thickness of dense planar crystals is formed at the bond between the single-layer Fe60 alloy powder coating and the substrate. The solidification organization of the Fe60 alloy coating consists of planar crystals with bright white bands, coarse cytosolic/columnar crystals, disordered and dense dendritic crystals, and equiaxed crystals in descending order from the bottom to the top.

(3) With a lap rate of 50%, the multi-channel Fe60 alloy powder coating quality is the best, and the coating lap rate area is smooth and uniform, which is a good combination, and no cracks, residue, or other defects are produced.

(4) The single-layer Fe60 alloy powder coating under the optimal process parameters shows better mechanical properties. The average microhardness of the Fe-based fused layer is 742 HV, which is 1.8 times that of the mixer liner; in the friction and wear test under the same parameters, the average friction coefficient of the Fe60 alloy powder coating is 0.57, the average friction coefficient of the substrate is 0.64, the wear quality between the surface of the mixer liner and the single-layer Fe60 alloy powder coating is 0.7 mg and 0.5 mg, respectively, and the wear quality of the surface of the mixer liner is significantly reduced by laser melting. 

## Figures and Tables

**Table 1:** Main chemical components of mixer lining plate.

Elements	C	Si	Mn	P	Ni	Mo	S	Fe
Content (Wt.%)	3.76	0.53	0.35	0.018	0.012	<0.01	0.027	Bal

**Table 2:** Chemical composition of Fe60 alloy powder.

Elements	C	Si	B	Cr	Ni	Fe
Content (wt.%)	0.8–1.2	1.0–2.0	3.8–4.2	16–18	9.0–12	Bal

**Table 3:** Main parameters of the laser cladding device.

Parameter Name	Parameter Data
Wavelength range	800–1100 nm
Power adjustment range	10–100%
Laser power	0~4000 W
Power density	104–1010 W/cm^2^
Maximum modulation frequency	5 kHz

**Table 4:** Mesh element size parameters.

Region	Maximum Element Size (mm)	Minimum Element Size (mm)
Upper	0.45	0.0849
Middle	2	0.36
Lower	10	1.4

**Table 5:** Thermo-physical parameters of mixer lining plate.

Temperature (K)	Density (kg/m^3^)	Specific Heat (J/kg/K)	Thermal Conductivity (W/m/K)
300	7716	573	11.9
600	7643	626	16.3
900	7548	728	19.4
1200	7420	705	21.6
1500	7015	753	31.6

**Table 6:** Thermophysical parameters of Fe60 alloy powder.

Temperature (K)	Density (kg/m^3^)	Specific Heat (J/kg/K)	Thermal Conductivity (W/m/K)
300	7580	480	18.1
600	7490	590	23.2
900	7380	840	25.9
1200	7340	740	27.5
1500	7155	845	30.7

**Table 7:** Parameters used in calculations.

Parameters	Symbol	Value	Unit
Initial temperature	$T_{0}$	293.15	K
Convective heat transfer coefficient	$h_{c}$	1250	W/m^2^/K
Stephen–Boltzmann constant	σ	5.67 × 10^−8^	W/m^2^/K^4^
Surface heat dissipation rate	ε	0.8	
Laser absorption rate	α	0.4	
Laser masking rate	β	0.5	
Laser power	P	900	W
Spot radius	$r_{0}$	1.5	mm
Scanning speed	v	600	mm/min
Powder feeding speed	φ	7	g/min
Powder feeding speed coefficient	τ	2.7 × 10^−3^	
Radius of the powder flow	$r_{m}$	1.5	mm

**Table 8:** Orthogonal test factors and levels.

Controlling Factors	Laser Power (W)	Scanning Speed (mm/min)	Powder Feeding Speed (g/min)
1	800	400	7
2	900	500	9
3	1000	600	11

**Table 9:** Orthogonal test table.

Specimen Number	Laser Power (W)	Scanning Speed (mm/min)	Powder Feeding Speed (g/min)
1	800	400	7
2	800	500	9
3	800	600	11
4	900	400	9
5	900	500	11
6	900	600	7
7	1000	400	11
8	1000	500	7
9	1000	600	9

**Table 10:** Microhardness score of cladding layer.

Interval (HV)	550–599	600–649	650–699	700–749	750–800
Score	0–2	3–4	5–6	7–8	9–10

**Table 11:** Surface quality rating table of cladding layer.

Serial Number	Surface Morphology of the Cladding Layer	Score
1	The surface of the coating is flat, is bright, has no air holes, and has no cracks; wetting angle θ ($\theta <90^{\circ }$)	9–10
2	The coating surface is flat, is bright, is microporous, and has no cracks; wetting angle θ ($\theta <90^{\circ }$)	7–8
3	The coating surface is flat, is bright, is microporous, has a small number of cracks; wetting angle θ ($90^{\circ }\leq \theta <120^{\circ }$)	5–6
4	The coating surface is rough, has obvious pores, and has a small number of cracks; wetting angle θ ($\theta \geq 120^{\circ }$)	3–4
5	The coating surface is rough, has a large number of pores, and has a large number of cracks; wetting angle θ ($\theta \geq 120^{\circ }$)	0–2

**Table 12:** Rating table of dilution rate of cladding layer.

Interval (%)	0–9	10–20	21–30	31–40	41–50	51–60
Score	0–2	9–10	7–8	5–6	3–4	0–2

**Table 13:** Surface quality table of cladding layer.

Specimen Number	Macro-Image	Microscopic Cross-Section Morphology
1	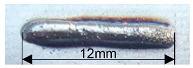	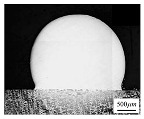
2	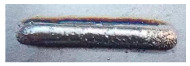	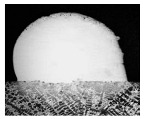
3	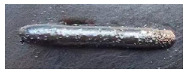	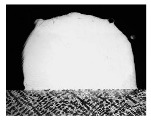
4	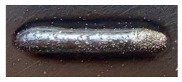	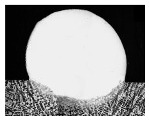
5	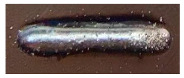	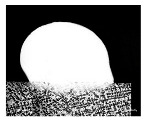
6	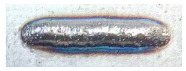	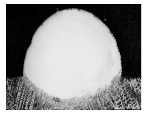
7	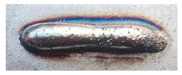	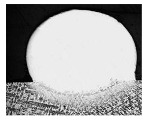
8	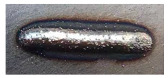	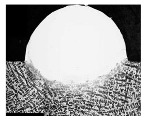
9	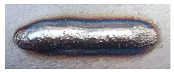	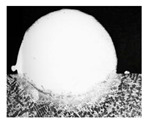

**Table 14:** Calculation results of coating dilution rate and microhardness.

Specimen Number	Melting Width (mm)	Melting Depth (mm)	Melting Height (mm)	Dilution Ratio (%)	Microhardness (HV)
1	1.751	0.036	1.511	2.3	663
2	1.759	0.062	1.311	4.5	661
3	1.717	0.039	1.523	2.5	626
4	2.131	0.516	1.606	24	726
5	1.825	0.061	1.655	3.5	647
6	2.023	0.456	1.048	30	767
7	2.129	0.665	1.887	26	683
8	2.305	0.597	1.190	33	730
9	2.047	0.470	1.261	27	738

**Table 15:** Results of weighted comprehensive analysis.

Specimen Number	Surface Hardness	Dilution Ratio	Surface Quality	Comprehensive Evaluation Score
1	5	0	5	3.5
2	5	0	5	3.5
3	4	0	5	3
4	8	8	6	7.6
5	4	0	5	3
6	9	7	9	8.4
7	6	7	6	6.3
8	8	6	9	7.6
9	8	7	9	7.9

**Table 16:** Process parameters of cladding layers with different bonding rates.

Specimen Number	Laser Power (W)	Scanning Speed (mm/min)	Powder Feeding (g/min)	Overlap Rate (%)
1	900	600	7	30
2	900	600	7	40
3	900	600	7	50

## Data Availability

The original contributions presented in the study are included in the article, further inquiries can be directed to the corresponding author.
